# Pomegranate Polyphenols Lower Lipid Peroxidation in Adults with Type 2 Diabetes but Have No Effects in Healthy Volunteers: A Pilot Study

**DOI:** 10.1155/2013/708381

**Published:** 2013-07-10

**Authors:** Arpita Basu, Emily D. Newman, Alecia L. Bryant, Timothy J. Lyons, Nancy M. Betts

**Affiliations:** ^1^Department of Nutritional Sciences, College of Human Sciences, 301 Human Sciences, Oklahoma State University, Stillwater, OK 74078-6141, USA; ^2^Section of Endocrinology and Diabetes, University of Oklahoma Health Sciences Center, Oklahoma City, OK 73104, USA

## Abstract

*Aims*. To examine the antioxidant and anti-inflammatory effects of pomegranate polyphenols in obese patients with type 2 diabetes (T2DM) (*n* = 8) and in healthy nondiabetic controls (*n* = 9). *Methods*. Participants received 2 capsules of pomegranate polyphenols (POMx, 1 capsule = 753 mg polyphenols) daily for 4 weeks. Blood draws and anthropometrics were performed at baseline and at 4 weeks of the study. *Results*. Pomegranate polyphenols in healthy controls and in T2DM patients did not significantly affect body weight and blood pressure, glucose and lipids. Among clinical safety profiles, serum electrolytes, renal function tests, and hematological profiles were not significantly affected by POMx supplementation. However, aspartate aminotransferase (AST) showed a significant increase in healthy controls, while alanine aminotransferase (ALT) was significantly decreased in T2DM patients at 4 weeks (*P* < 0.05), though values remained within the normal ranges. Among the biomarkers of lipid oxidation and inflammation, oxidized LDL and serum C-reactive protein (CRP) did not differ at 4 weeks in either group, while pomegranate polyphenols significantly decreased malondialdehyde (MDA) and hydroxynonenal (HNE) only in the diabetic group versus baseline (*P* < 0.05). *Conclusions*. POMx reduces lipid peroxidation in patients with T2DM, but with no effects in healthy controls, and specifically modulates liver enzymes in diabetic and nondiabetic subjects. Larger clinical trials are merited.

## 1. Introduction

Among the fruits with demonstrated cardiovascular benefits, the pomegranate *(Punica granatum L.)* has gained significant attention in complementary and alternative health practices. Pomegranate juice has been rated to possess the highest antioxidant capacity when compared to the commonly consumed polyphenol-rich beverages in the United States [1]. Several categories of phytochemicals are present in the pomegranate including flavonoids (flavonols, flavanols, and anthocyanins), condensed tannins (proanthocyanidins), and hydrolysable tannins (ellagitannins and gallotannins). Additional phytochemicals present in pomegranates include organic and phenolic acids, sterols and triterpenoids, and alkaloids. The seeds of the pomegranate are rich in crude fibers, pectin, and sugars [2, 3]. Thus, pomegranate polyphenols in cell culture, animal model systems, and limited clinical research have been reported to exert several vascular benefits, including antioxidant and anti-inflammatory effects [2, 3]. Pomegranate juice supplementation has been shown to ameliorate hypertension and reduce surrogate risk factors of atherosclerosis in a few clinical studies [4–8]. However, the safety as well as efficacy of pomegranate polyphenol extracts, especially as commercially available dietary supplements in the United States, needs further investigation in healthy subjects as well as in those with cardiovascular risk factors.

Obesity and type 2 diabetes (T2DM) are significant public health problems in the United States and have been associated with several modifiable factors, including dietary selection of foods and beverages. The role of nutraceuticals, including pomegranate polyphenols, in the therapeutic management of these conditions has been identified in the reported literature, though the evidence is largely based on studies in cell culture and animal models [9, 10]. In a single clinical study reported by Heber et al. (2007) in overweight individuals, ellagitannin-enriched pomegranate polyphenol extracts were shown to be well tolerated and decrease plasma thiobarbituric acid reactive substances (TBARS), a biomarker of lipid oxidation at 4 weeks of supplementation [11]. However, this study excluded subjects with diabetes and hypertension and did not investigate the effects of pomegranate polyphenols on other notable biomarkers of oxidative stress and inflammation, such as oxidized LDL and C-reactive protein (CRP) [11]. Thus, as highlighted in a recently published review by Medjakovic and Jungbauer (2013), the role of pomegranates in reducing cardiovascular risks needs to be further examined and clarified in clinical studies [10]. Thus, the present pilot clinical study was conducted to examine the effects of a 4-week supplementation of pomegranate polyphenol extracts (POMx) on body weight, blood pressure, clinical variables, and biomarkers of lipid oxidation and inflammation in obese adults with type 2 diabetes and in healthy controls. 

## 2. Materials and Methods

### 2.1. Study Design and Subjects

This was a 4-week pre- and postintervention study investigating the effects of pomegranate polyphenol supplementation (POMx capsules, Pom Wonderful, CA, USA), in subjects with T2DM and in healthy volunteers. This study was conducted according to the guidelines presented in the Declaration of Helsinki and approval was obtained from the Institutional Review Board (IRB) at the Oklahoma State University (OSU) for all procedures. All participants provided a signed informed consent prior to enrollment in the study. Subjects were recruited at the Department of Nutritional Sciences at OSU through campus e-mail advertisements and flyer. Participants in the diabetic group (T2DM) were required to meet the following criteria: diagnosis of type 2 diabetes as defined by the American Diabetes Association (ADA) [12], abdominal adiposity (waist circumference >35 inches in women and >40 inches in men), and stable on oral hypoglycemic agents. Healthy controls were recruited based on a waist circumference of less than 35 inches for women and less than 40 inches for men, and without any diagnosis of chronic disease, such as diabetes, cancer, or any other form of cardiovascular disease. Subjects in both groups were excluded if they were pregnant, nursing, taking mega doses of antioxidants/fish oil supplements (>1 g/day), and having an abnormal hematological profiles, abnormal liver, kidney, and thyroid function tests. Individuals who smoked or used any other form of tobacco were excluded as well as those who consumed alcohol on a daily basis. 

The participants were supplemented with a daily dose of two POMx capsules (1 capsule = 753 mg polyphenols) for a period of four weeks. The POMx capsules were purchased from Pom Wonderful LLC (Los Angeles, CA, USA).  Table 1 shows the composition of the two pomegranate extract capsules administered to the study participants. Total phenolics and total ellagic acid content were determined by Brunswick Laboratories (Norton, MA, USA). Contents of moisture, ash, protein, fat, and carbohydrate were determined by the Robert M. Kerr Food and Agriculture Products Center, OSU (Stillwater, OK, USA). The participants were asked to consume 2 capsules, one in the morning and one in the evening with water only.

Participants were asked to restrict intake of commonly consumed polyphenol-containing foods for 2 weeks prior to the start of the intervention. Blood draws and measures of blood pressure and anthropometrics (height, weight, and waist circumference) were obtained at the initial screening visit (week 0) and at end of the study (week 4) for both diabetic patients and healthy controls. Subjects were asked to maintain their usual diet, physical activity, and lifestyle during the study. Subjects were instructed to complete detailed 3-day food records during the first and the fourth weeks of the study. Participants were instructed on how to use the food records and also how to accurately record food portions consumed. Venipuncture was conducted by a certified phlebotomist at the Stillwater Medical Center (Stillwater, OK, USA) and plasma and serum samples were separated and stored at −80°C for future analyses. Compliance was assessed using pill counts and plasma ellagic acid measured using a previously published procedure [13].

### 2.2. Blood Pressure

Systolic and diastolic blood pressure measurements were obtained using a portable blood pressure device with arm cuff, Spot Vital Signs Device (Welch Allyn, Skaneateles Falls, NY, USA). The average of three measurements was taken at an interval of 5–7 minutes.

### 2.3. Anthropometrics

The Health-o-Meter Weight Tracking Scale (Sunbeam Products, Inc., Maitland, FL, USA) was used to determine the participant's body weight and the Gulick II tape measure (Vital Signs, Gay Mills, WI, USA) was used to measure waist circumference at the super iliac crest in inches. Height was measured without shoes by using the Accustat Genentech Stadiometer (San Francisco, CA, USA), and height was recorded to the nearest 0.1 cm. 

### 2.4. Clinical Variables

Freshly drawn blood samples from baseline and week four of the study were analyzed for glucose, lipids, insulin, HbA1c, CRP, safety profiles including serum proteins, electrolytes, liver, kidney, and thyroid function tests, and hematology at the Stillwater Medical Center (Stillwater, OK, USA). Serum oxidized LDL was measured in triplicate using an oxidized LDL competitive ELISA (Mercodia, Uppsala, Sweden) and serum malondialdehyde and 4-hydroxynonenal (MDA and HNE) were determined with the Bioxytech LPO-586 assay (OxisResearch Inc., Foster City, CA, USA). The interassay coefficient of variation was within 8–10% for these assays. 

### 2.5. Dietary Analysis

Participants' 3-day food records were used to examine any dietary changes during the course of the 4-week study. Food record analyses were performed using ESHA Food Processor version 9.1.0 (ESHA Research Inc., Salem, OR, USA). The average of 3-day intakes was calculated at baseline and week 4 of the study.

### 2.6. Statistical Analysis

Descriptive statistics were calculated and data graphed for outliers. Data have been reported as means ± standard deviations. Our primary comparison was the differences in means at 0 and 4 weeks of the study within each group of T2DM and healthy non-diabetic controls. Thus, paired *t*-tests were performed to determine differences between pre- (week 0) and postintervention (week 4) values within each group. All variables were normally distributed and no transformation was used. Statistical significance was set at *P* < 0.05 (two-sided test) and all data analysis was performed with SPSS version 16.0 (SPSS Inc., Chicago, IL, USA).

## 3. Results

Table 1 shows the nutritional composition of the POMx supplementation. We found a lower content of polyphenols per capsule in comparison to the label claim of 1000 mg total polyphenols by the manufacturers (Pom Wonderful, LA, CA, USA). The POMx capsules were also a significant source of ellagic acid and carbohydrates, but a negligible source of fats and proteins.  Table 2 shows the baseline characteristics of our study subjects. All nine healthy controls in our study had an overall healthy BMI (<25), waist circumference less than 35 inches, not on any medications for hypertension and diabetes, but a significant proportion was using multivitamin and/or mineral supplements on a regular basis. On the other hand, among patients with T2DM, two out of eight were males, prevalence of obesity was high as assessed by BMI (>30) and waist circumference, 75% of patients were controlling their glucose levels with oral hypoglycemic agents and the remaining with diet and exercise, and 50% were using multivitamin and/or mineral supplements on a regular basis. None of our patients with T2DM were on insulin therapy. Pill count showed 100% compliance and plasma ellagic acid was detectable in approximately 85% of our participants at 4 weeks of intervention. 

As shown in Table 3, POMx supplementation did not significantly affect glucose, HbA1c, blood pressure, and lipids at 4 weeks versus baseline in healthy controls as well as in T2DM patients.  Table 4 lists the safety profiles measured in our study participants which were not significantly affected by POMx supplementation, except a small but significant increase in aspartate aminotransferase (AST) in healthy controls, and also a small but significant decrease in alanine aminotransferase (ALT) in T2DM patients at 4 weeks versus baseline (*P* < 0.05). However, values of AST and ALT remained within the normal clinical range defined in our study at baseline and at 4 weeks (AST: 14–36 U/L; ALT: 7–56 U/L). 

**Figure 1:**
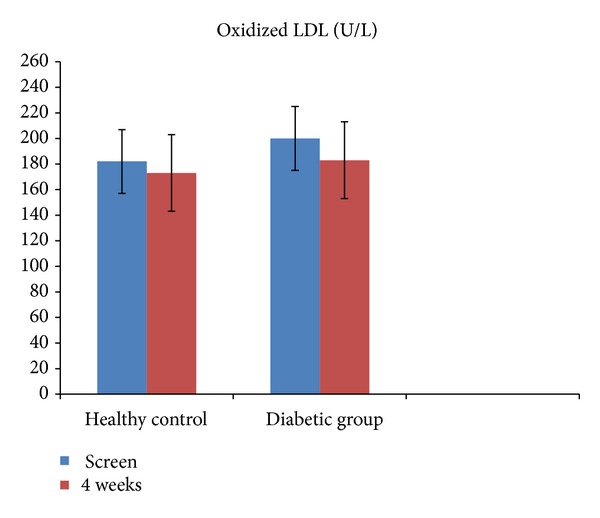
Serum oxidized LDL in healthy controls (*n* = 9) and in patients with type 2 diabetes (*n* = 8) before and after (4 weeks) pomegranate polyphenol supplementation. Data presented as mean ± SD.

**Figure 2:**
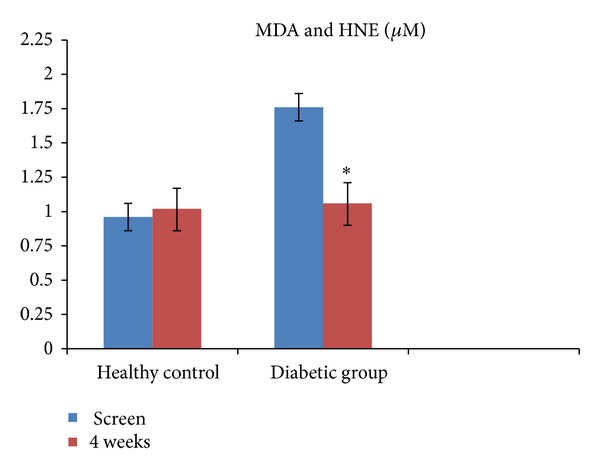
Serum malondialdehyde (MDA) and hydroxynonenal (HNE) in healthy controls (*n* = 9) and in patients with type 2 diabetes (*n* = 8) before and after (4 weeks) pomegranate polyphenol supplementation. Data presented as mean ± SD. *Significantly different from baseline at *P* < 0.05.

**Figure 3:**
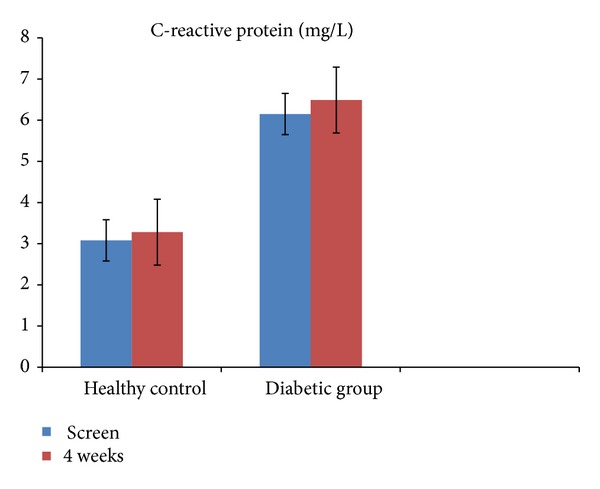
Serum C-reactive protein in healthy controls (*n* = 9) and in patients with type 2 diabetes (*n* = 8) before and after (4 weeks) pomegranate polyphenol supplementation. Data presented as mean ± SD.

 Among the biomarkers of lipid oxidation, no significant differences were noted in oxidized LDL (Figure 1), while MDA and HNE were significantly decreased at 4 weeks only in T2DM patients following pomegranate polyphenol supplementation (Figure 2; *P* < 0.05). These results remained significant when adjusted for the proportion of multivitamin users in the diabetic group. However, pomegranate polyphenol supplementation did not significantly affect CRP, a stable biomarker of inflammation in healthy controls as well as in T2DM patients (Figure 3).

 No significant differences in dietary intakes of macro- and micronutrients were noted at baseline and 4 weeks of the intervention in healthy controls as well as in T2DM patients (Table 5). 

## 4. Discussion

Our study findings in healthy volunteers and in patients with T2DM without complications provide evidence on the safety and efficacy of a 4-week pomegranate polyphenol extract supplementation. Our pre- and postintervention data analyses show that POMx supplementation (2 capsules/day ~1500 mg polyphenols) did not significantly alter safety parameters in both groups of participants. However, we observed a specific modulation of liver enzymes (AST and ALT) at 4 weeks versus baseline, though values remained within the normal clinical range defined in our study. POMx supplementation did not affect body weight, blood pressure, glucose, and lipids in healthy controls as well in T2DM patients. Among the biomarkers of lipid oxidation and inflammation, POMx supplementation significantly reduced MDA and HNE only in T2DM patients, while oxidized LDL and serum CRP were not significantly affected at 4 weeks in either group. Overall, our study findings show modest effects of POMx supplementation in improving lipid peroxidation, only in diabetic patients versus baseline.

 Type 2 diabetes (T2DM) is an overwhelming public health concern and has been significantly associated with elevated lipid oxidation and inflammation [14]. Observational data derived from the National Health Interview Survey (NHIS) in the United States reveal a high prevalence of nonvitamin/nonmineral supplement use among adults with T2DM, but no significant associations with disease severity [15]. These observations identify the urgent need for studies to examine the efficacy of dietary supplements in improving clinical outcomes of T2DM, especially supplements containing botanical ingredients, with claims in health and disease. In case of pomegranate supplements, clinical studies, though few, report some promising data on their role in the therapeutic management of T2DM. In an uncontrolled study for 4–6 weeks, pomegranate juice and concentrates (~650 mg gallic acid equivalents) were shown to significantly decrease TBARS and increase serum thiols, thereby showing an improvement in oxidative stress in diabetic patients. Though pomegranate intervention in this study showed no effects in glucose and lipid levels, the activity of paraoxonase 1 (PON1) associated with the antiatherosclerotic activities of HDL was significantly increased following pomegranate supplementation [16]. Similar antioxidant effects of pomegranate juice in diabetic patients have also been reported by other studies conducted by the same group of researchers in Israel [6]. In another study reported from Iran in T2DM patients with hyperlipidemia, concentrated pomegranate juice supplementation (40 g for 8 weeks) was shown to decrease total and LDL-cholesterol, but not HDL-cholesterol and triglycerides versus baseline [17]. Thus, our study findings of significant lowering of lipid peroxidation with a 4-week POMx supplementation (2 capsules/day) in diabetic patients, but with no effects on glucose and lipids, conform to some of these previous clinical studies. However, it should be noted that all of these studies, including ours, were conducted using a small sample size with no placebo-controlled group. In a single placebo-controlled study of T2DM patients, a 3-month antioxidant supplementation of a combination of pomegranate and green tea extracts and ascorbic acid was shown to significantly reduce LDL-cholesterol, increase HDL-cholesterol, and decrease lipid peroxidation versus placebo [18]. However, the study did not report safety profiles following antioxidant supplementation and does not identify the effects of pomegranate polyphenols *per se*. 

 The use of botanical supplements with health and disease claims has been an issue of safety concerns. Researchers have identified toxicities associated with dietary supplement use as a result of deviations from good manufacturing practices, including contamination with heavy metals and microbes, as well as toxic effects of unidentified constituents in the supplements [19]. Pomegranate polyphenol extracts (~1400 mg) have been reported to be safe in a single randomized placebo-controlled study reported by Heber et al. (2007) in overweight subjects [11], and pomegranate juice supplementation (~8 oz) and higher dose of extracts (~3000 mg) have also shown no adverse effects in phase II clinical trials in patients with prostate cancer [20, 21]. However no such studies on clinical safety profiles have been reported in patients with T2DM. Thus, our novel findings show no significant adverse effects of two capsules of POMx (~1500 mg polyphenols) for 4 weeks in obese patients with T2DM. We observed a small but significant decrease in hepatic ALT at 4 weeks though values remained within the normal clinical range used in our study. Keeping in view the short duration of our intervention, larger placebo-controlled dose-response studies of longer duration are needed to confirm the effects of pomegranate polyphenols in T2DM patients with and without complications.

Few studies have examined the effects of pomegranate polyphenols in nonobese healthy volunteers with no history of chronic diseases. Pomegranate juice supplementation for 4 weeks has been reported to significantly reduce systolic and diastolic blood pressure [22] and suppress high-fat meal-induced postprandial rise in systolic blood pressure [23], versus control groups in healthy volunteers. Interestingly, the amount of pomegranate polyphenols administered in these studies [22, 23] was lower than the dose used in our study. Furthermore, in contrast to these previous studies, we did not observe any significant changes in systolic and diastolic blood pressure in our healthy volunteers at 4 weeks of pomegranate polyphenol intervention. These discrepancies might be explained by the sample size, dose and form of delivery of pomegranate polyphenols (juice versus capsules), and duration of the studies. 

Our study has some limitations including a small sample size of healthy volunteers and T2DM patients and the absence of dose-response effects and a no treatment control group. In addition, we were unable to detect gender-wise differences in our small study sample, and this would be of specific relevance to lipid profiles following intervention. Further, we did not include T2DM patients with complications, or those on insulin therapy. Thus, our study in well-controlled T2DM patients with no complications cannot be generalized to the larger diabetic population. 

## 5. Conclusion

We conclude that commercially available pomegranate polyphenols (POMx 1500 mg/day for 4 weeks, Pom Wonderful, CA, USA) were well tolerated with no adverse effects in healthy volunteers as well as in patients with type 2 diabetes. In addition, POMx lowered lipid peroxidation only in diabetic patients but had no effects on glucose, lipids, and C-reactive protein in either group of participants.

## Figures and Tables

**Table 1:** Composition of POMx capsules^1^.

Component (weight)	Weight
Carbohydrates (mg)	1801
Hexane extract (fat) (mg)	5.86
Protein (mg)	72.5
Ash (mg)	112.6
Moisture (mg)	116.08
Total ellagic acid (mg)	372
Total phenolics (mg)*	1505.28

^1^Data presented per 2101 mg (2 capsules). Source: Pom Wonderful (Los Angeles, CA, USA).

*Expressed as mg gallic acid equivalents.

**Table 2:** Baseline characteristics of healthy controls and subjects with type 2 diabetes (T2DM).

Characteristic	Healthy controls	T2DM subjects
Gender		
Male/female (*n*/*n*)	0/9	2/6
Age (mean, SD)	47.1, 6.3	52.4, 13.3
Weight kg (mean, SD)	62.6, 10.3	99.3, 29.1
Height cm (mean, SD)	163.7, 6.0	167.6, 7.2
BMI kg/m^2^	23.3, 3.0	35.3, 10.3
Waist circumference inches (mean, SD)	31.06, 2.3	42.4, 7.9
Supplement use (%)	7 (78%)	4 (50%)
Vitamin/mineral (%)	7 (78%)	4 (50%)
Herb or botanical (%)	0 (0%)	0 (0%)
Antioxidant in the past (%)	2 (22%)	1 (13%)
Fish oil (%)	1 (11%)	1 (13%)
Blood pressure medication (%)	0 (0%)	2 (25%)
Diabetic medication (%)	0 (0%)	6 (75%)
Diabetes duration (years)	N/A	3.5
Aspirin use (%)	0 (0%)	1 (13%)
Other medications (%)	1 (11%)	6 (75%)

**Table 3:** Effects of POMx on blood glucose, blood pressure, and lipids^1^.

Variables	Healthy controls (*n* = 9)		T2DM subjects (*n* = 8)	
	Screen	Week 4	Screen	Week 4
Fasting glucose (mg/dL)	85.11 ± 4.48	85.67 ± 8.03	105.88 ± 29.57	114.25 ± 33.36
Mean blood glucose (mg/dL)	97.33 ± 16.93	97.33 ± 19.01	121.25 ± 16.73	116.38 ± 11.56
Hemoglobin A1C (%)	5.51 ± 0.51	5.51 ± 0.57	6.22 ± 0.50	6.08 ± 0.35
Glycosylated hemoglobin (%)	6.45 ± 0.76	6.45 ± 0.85	7.51 ± 0.75	7.31 ± 0.53
Insulin (U/L)	7.77 ± 2.44	10.52 ± 12.06	18.61 ± 7.30	19.06 ± 6.03
Systolic blood pressure (mmHg)	114.67 ± 6.18	115.33 ± 5.81	129.00 ± 18.19	134.25 ± 23.81
Diastolic blood pressure (mmHg)	71.33 ± 7.37	72.67 ± 9.25	81.63 ± 8.25	79.88 ± 5.17
Total cholesterol (mg/dL)	187.89 ± 30.16	195.56 ± 30.53	182.13 ± 40.47	181.50 ± 36.73
Triglycerides (mg/dL)	77.11 ± 18.43	77.56 ± 26.88	149.75 ± 94.73	135.63 ± 66.06
LDL-cholesterol (mg/dL)	109.22 ± 27.87	117.22 ± 30.05	87.50 ± 43.09	103.75 ± 27.42
HDL-cholesterol (mg/dL)	63.11 ± 7.56	62.89 ± 9.35	51.75 ± 11.45	50.38 ± 11.03
VLDL-cholesterol (mg/dL)	15.33 ± 3.67	15.44 ± 5.29	30.00 ± 19.04	27.25 ± 13.32
LDL/HDL ratio	1.76 ± 0.52	1.93 ± 0.66	1.98 ± 0.68	2.15 ± 0.75
Total cholesterol/HDL ratio	2.89 ± 0.78	3.05 ± 0.88	3.63 ± 0.92	3.58 ± 0.96

^1^Data are mean ± SD; T2DM: type 2 diabetes mellitus.

**Table 4:** Effects of POMx supplementation on safety parameters^1^.

Variables	Healthy controls (*n* = 9)		T2DM subjects (*n* = 8)	
	Screen	Week 4	Screen	Week 4
Aspartate aminotransferase (U/L)	21.78 ± 4.29	25.33 ± 6.82*	30.00 ± 9.71	30.75 ± 9.42
Alanine aminotransferase (U/L)	25.33 ± 8.23	27.22 ± 11.94	36.88 ± 13.47	33.13 ± 10.05*
Alkaline phosphatase (U/L)	72.89 ± 8.23	74.22 ± 24.79	91.50 ± 17.96	87.50 ± 11.07
Bilirubin, total (mg/dL)	0.43 ± 0.26	0.39 ± 0.24	0.33 ± 0.10	0.43 ± 0.18
Total protein (g/dL)	6.94 ± 0.32	6.96 ± 0.38	7.15 ± 0.33	7.23 ± 0.44
Albumin (g/dL)	4.18 ± 0.31	4.20 ± 0.25	4.14 ± 0.32	4.19 ± 0.32
Globulin (g/dL)	2.78 ± 0.29	2.76 ± 0.22	3.00 ± 0.21	3.05 ± 0.21
Albumin/globulin ratio	1.51 ± 0.20	1.53 ± 0.14	1.40 ± 0.17	1.38 ± 0.12
Sodium (mEq/L)	139.78 ± 1.79	139.89 ± 2.67	140.38 ± 3.02	140.50 ± 2.73
Potassium (mEq/L)	4.28 ± 0.42	4.21 ± 0.31	4.25 ± 0.18	4.25 ± 0.37
Chloride (mEq/L)	106.56 ± 2.60	107.56 ± 1.67	105.00 ± 1.20	105.50 ± 1.31
Calcium (mg/dL)	9.60 ± 0.46	9.40 ± 0.44	9.51 ± 0.52	9.49 ± 0.39
Thyroxine (T4) (ug/dL)	6.91 ± 1.55	6.42 ± 1.47	8.14 ± 2.20	7.70 ± 1.55
T3 Uptake (%)	31.22 ± 3.31	31.56 ± 3.48	29.10 ± 1.70	30.79 ± 2.75
Blood urea nitrogen (mg/dL)	12.11 ± 2.67	13.00 ± 4.12	15.25 ± 3.92	15.50 ± 2.93
Creatinine (mg/dL)	0.82 ± 0.20	0.80 ± 0.15	0.83 ± 0.18	0.86 ± 0.18
Blood urea nitrogen/creatinine ratio	14.93 ± 2.72	16.28 ± 5.01	18.39 ± 2.71	18.64 ± 5.40
White blood cell (K/mm^3^)	5.17 ± 0.77	5.09 ± 1.18	7.89 ± 2.01	7.40 ± 1.80
Red blood cell (M/mm^3^)	4.45 ± 0.32	4.49 ± 0.33	4.78 ± 0.25	4.74 ± 0.28
Hemoglobin (gm/dL)	13.74 ± 0.90	13.86 ± 0.91	13.69 ± 0.69	13.58 ± 0.71
Hematocrit (%)	40.87 ± 2.82	40.92 ± 2.51	41.23 ± 2.30	40.84 ± 2.28
Platelet count (K/mm^3^)	271.22 ± 59.16	269.78 ± 63.44	259.00 ± 70.07	276.13 ± 55.24

^1^Data are mean ± SD.

*Significantly different from baseline (*P* < 0.05); T2DM: type 2 diabetes mellitus.

**Table 5:** Dietary intakes^1,2^.

Nutrient	Healthy controls (*n* = 9)		T2DM subjects (*n* = 8)	
	Screen	Week 4	Screen	Week 4
Energy (kcal)	1679.42 ± 285.13	1739.05 ± 462.52	1266.32 ± 413.65	1273.38 ± 412.62
Protein (g)	70.71 ± 10.48	62.14 ± 15.29	60.74 ± 11.70	70.95 ± 23.45
Carbohydrate (g)	213.00 ± 45.42	254.90 ± 113.23	140.59 ± 50.55	129.42 ± 51.92
Fiber (g)	22.53 ± 5.33	19.75 ± 7.11	13.46 ± 4.83	12.11 ± 3.64
Total fat (g)	65.94 ± 16.21	55.62 ± 21.71	53.64 ± 22.81	54.98 ± 20.18
Saturated fat (g)	19.07 ± 6.40	15.74 ± 8.85	15.09 ± 7.05	18.91 ± 6.53
Monounsaturated fat (g)	17.18 ± 6.37	14.90 ± 7.80	13.71 ± 8.25	14.54 ± 7.11
Polyunsaturated fat (g)	12.36 ± 5.32	9.00 ± 5.03	9.91 ± 6.68	6.90 ± 5.07
Cholesterol (mg)	180.57 ± 69.85	144.51 ± 65.79	256.11 ± 179.52	236.83 ± 164.64
Carotenoids (RE)	773.01 ± 443.40	434.37 ± 240.67	486.79 ± 294.38	428.45 ± 207.05
Vitamin C (mg)	123.30 ± 55.90	278.77 ± 453.17	69.25 ± 44.85	51.61 ± 36.33
Vitamin E (mg)	6.66 ± 4.97	3.63 ± 2.36	5.05 ± 4.60	4.21 ± 5.46
Copper (mg)	0.99 ± 0.32	0.78 ± 0.40	0.60 ± 0.23	0.59 ± 0.23
Iron (mg)	13.82 ± 3.87	12.95 ± 3.06	9.85 ± 4.24	12.11 ± 6.02
Zinc (mg)	8.39 ± 2.24	7.30 ± 2.43	4.93 ± 1.85	7.43 ± 2.99

^1^Data are mean ± SD.

^2^Data summarized from 3-day food records; T2DM: type 2 diabetes mellitus.
